# Note from the Editor: Oil Spill Research Update

**Published:** 2010-10

This fall, the National Institutes of Health (NIH) will launch a multiyear epidemiologic study to investigate effects of the *Deepwater Horizon* oil spill on human health. The project, led by the National Institute of Environmental Health Sciences, will track the health status of workers and volunteers who contributed to oil spill clean-up, focusing on potential effects of exposure to oil and dispersants.

